# Health promotion initiative: A dementia-friendly local community in Sweden

**DOI:** 10.1177/1471301220977736

**Published:** 2020-12-08

**Authors:** Elzana Odzakovic, Ingrid Hellström, Ann-Charlotte Nedlund, Agneta Kullberg

**Affiliations:** 4566 Department of Nursing, School of Health and Welfare, 4161 Jönköping University, Jönkoping, Sweden; Division of Nursing Science and Reproductive Health, Department of Health, Medicine and Caring Sciences, The Faculty of Medicine and Health Sciences, 4566 Linköping University, Sweden; 531597 Ersta Sköndal Bräcke University College, Stockholm, Sweden; Division of Society and Health, Department of Health, Medicine and Caring Sciences, The Faculty of Medicine and Health Sciences, 4566 Linköping University, Sweden; Division of Society and Health, Department of Health, Medicine and Caring Sciences, The Faculty of Medicine and Health Sciences, 4566 Linköping University, Sweden

**Keywords:** public health, dementia, experiences, health promotion, dementia-friendly community, qualitative interviews, awareness

## Abstract

Dementia is a great public health concern worldwide. Despite this, little is known from a health-promoting perspective about dementia in general as a public health issue, in dialog with people living with dementia, applicable at individual, group, and societal levels with regard to policies and practice. This study therefore aims to explore the experiences related to living with dementia in the local community by advancing a health-promoting perspective. Semi-structured individual and group interviews were conducted with participants (*n* = 22) with lived, professional, and personal experiences of dementia living in a medium-sized municipality in Sweden. Transcripts were analyzed by thematic analysis. Four themes emerged: health promotion through knowledge and public awareness, health promotion through opportunities to be active, health promotion through meaningful meeting places, and health promotion through improvements in the welfare system. We found that more knowledge and public awareness about dementia are needed to advance a health-promoting perspective and increase the prominence of dementia as a public health issue. Further research and policy need to focus more on how professionals in dementia care practice could be involved in promoting health and well-being for people with dementia.

## Introduction

A paradigm shift needs to be developed worldwide in line with the global action plan for the public health response to dementia. Attention must be drawn to health-promoting strategies in dialog with people living with dementia. These strategies must be applicable at individual, group, and societal levels (WHO, 2012). There are several key domains of public health practice. The biomedical approach is well established and applied in healthcare systems on a daily basis by all health professionals, with a focus on diagnosis, treatment of diseases, and prevention of risk factors for specific diseases (Wiesmann & Hannich, 2014). Another core domain of public health is health promotion, applying a holistic biopsychosocial view, so that health is to be seen as a resource in people’s everyday life, as stipulated by the WHO: “*health promotion is the process of enabling people to increase control over, and to improve, their health*” (WHO, 1986, p. 1). Further, the health promotion construct sheds light on the positive relationship between well-being and health and that perceived health and well-being are not necessarily connected to whether a person has a diagnosis or not, considering that health is dynamic, perceived, and maintained in a continuum (Eriksson, 2007; Haglund et al., 1996).

Health promotion improves and sustains a person’s ability to act and realize what is important in everyday life; health goes beyond a biomedical approach (Nordenfelt, 2017). Antonovsky (1996) added a salutogenic view, when he stipulated that problems and stress are present continuously in human life, and it is our ability to handle these and our reciprocal interaction with our social and physical environment that maintain and develop our health. Antonovsky coined the concept of a sense of coherence, which is the sum of the three inherent dimensions, comprehensibility, manageability, and meaningfulness, that together build our generalized resistance resources that help us to manage stressors. A strong sense of coherence contributes to staying healthy and feeling well, even when a disease or illness is present (Antonovsky, 1987).

The biomedical approach has been established in dementia care for some time. Medical knowledge dominates according to targeted interventions for people with a dementia diagnosis (i.e., pharmaceuticals), whereas health promotion interventions are directed at salutary conditions that create self-rated well-being, strengthening self-efficacy for the whole person or group (Antonovsky, 1996; Lindström & Eriksson, 2006). Beyond the biomedical approach, in recent years, a variety of psychosocial initiatives have been developed. Examples include meeting centers (Brooker et al., 2018) and care planning interventions (Orsulic-Jeras et al., 2016; Quinn et al., 2016) that are inclusive of the partnership that exists between a person living with dementia and their supporters. However, health promotion community strategies with a focus on how to stay as well as possible, even with a dementia diagnosis, are rare (Keady et al., 2012). Therefore, there is a need for reorientation toward resources and strategies that encourage people living with dementia to live well in the community (Keady et al., 2012; Swaffer, 2015). There is a promising development of dementia-friendly communities globally (Alzheimer’s Disease International, 2017, 2018). A dementia-friendly community is suggested to be *“a place or culture in which people with dementia and their carers are empowered, supported and included in society, understand their rights and recognize their full potential”* (Alzheimer’s Disease International, 2016, p. 10). But, much of the research into making communities dementia-friendly does not include the experiences of people living with dementia (Buckner et al., 2019; Ebert et al., 2019; Herbert & Scales, 2019) and from the perspectives of health promotion in relation to a life with dementia (Swaffer, 2014).

Many interventions targeting people living with dementia are still based on the problems and symptoms associated with a dementia diagnosis and focus on the disease or the carer’s perspective (Rahman & Swaffer, 2018; Swaffer, 2014). This indicates that people with dementia, or with lived experiences of dementia, could be coproducers of their health outcomes (Rahman & Swaffer, 2018). Dementia as a public health issue, which needs to be considered from both health promotion and preventive perspectives, is often neglected in public health policies or interventions compared with other diseases, such as diabetes mellitus (Sørensen et al., 2015) or cardiovascular diseases (Makenzius & Wamala, 2015).

Dementia needs more attention and public awareness, both nationally and locally, due to increasing prevalence. This is a challenge for healthcare systems and society as a whole. Thus, a shift from focusing on individual symptoms and difficulties connected to dementia to looking at people’s resources and what interventions might promote their health is warranted. Health-promoting interventions are based on empowerment strategies that create a supportive physical and social environment in a local context (Haglund et al., 1996) and thereby health promotion is not only a matter for the healthcare system but is also a concern for society’s wider policy areas (Kickbusch, 2003).

To design health-promoting strategies, we need to understand more about how people living with dementia want to promote their health and well-being and how they manage physical, mental, and emotional stressors, using a salutogenic perspective and a focus on the resources people have (Antonovsky, 1996). This study therefore aims to explore experiences related to living with dementia in the local community by advancing a health-promoting perspective and addresses one central question: “What promotes health and well-being for people living with dementia in their local community?”

## Methods

### Research design

A qualitative exploratory design was chosen because this was the most appropriate method of taking part in the experiences (Phillipson & Hammond, 2018; Polit & Beck, 2016) of dementia by those living or working with dementia on a day-to-day basis.

### Setting

This study was conducted in Norrköping, a medium-sized municipality in south-eastern Sweden, where a project “Dementia-friendly community—the Norrköping model” has been ongoing. There are currently estimated to be approximately 158,000 people living with dementia in Sweden (The National Board of Health and Welfare, 2014), where municipalities are responsible for home care services for older people (Szebehely & Trygdegård, 2012). The home care services commonly granted to older people can include help in the home, personal care, meals on wheels, or day care services, which are only granted for people with dementia. The day care service provides a day time meeting place where people with dementia can go and where different activities are available together with staff (Odzakovic et al., 2018; Måvall & Malmberg, 2007).

Norrköping municipality has 141,000 inhabitants. The most common level of education is postsecondary school, and relatively few inhabitants have university degrees compared with the national level (Office of the Municipal Executive Board, 2018). People aged 65 years and older make up 19% of the population, and there is a higher proportion of foreign-born inhabitants (26%) compared with the total for Sweden (24.1%) (persons who have foreign backgrounds are defined as persons who are foreign born or born in Sweden with two foreign-born parents) (Office of the Municipal Executive Board, 2018). During the 20th century, Norrköping experienced increasing problems with unemployment as well as social and economic vulnerability. This has affected mortality and morbidity rates; for example, cardiovascular disease rates are significantly higher in Norrköping than in other similar-sized cities in Sweden (Faresjö et al., 2019; Wennerholm et al., 2011). Because of this epidemiology and the fact that cardiovascular diseases and dementia are mutually associated, Norrköping was selected as the intervention setting due to the higher predicated cases of dementia. Woodward et al. (2018) argue that the development of dementia-friendly communities is highly motivated within communities where there is a higher prevalence of dementia.

### Participant recruitment

The recruitment of participants started in 2017 and lasted until 2018 with the aim of including various experiences related to living with dementia in the local community. This includes people with lived experiences of dementia who consider themselves to have dementia, those who have cared for a spouse and those who are working within care practices with professional experience or had personal experience of dementia on a daily basis, and the capacity to give informed consent. We contacted, visited, and informed social and healthcare professionals working in the municipality (home care services and day care centers), voluntary organizations, politicians, and the Swedish church for details of any possible participants. Potential participants were then contacted by email, personal meeting, or telephone if they wanted to participate in the study.

In total, 22 participants with lived, personal, and professional experiences related to dementia participated in the semi-structured individual or group interviews. Of those 22 participants (Table 1), 14 were women and eight were men, eight were people were living with dementia, four were carers, and 10 were professionals working as nursing staff, social workers, care managers, or third sector employees. Most were of pensionable age, but others were less than 65 years old and therefore employable.

**Table 1.** Overview of the participants and their experiences related to dementia.

Participant (fictitious name)	Semi-structured		Person diagnosed with dementia	Carer/professional/voluntary organization
	Individual interview (II)	Group interview (GI)		
Alice (employed)	X			X
Astrid (pensioner)	X		X	
Beatrice (employed)	X			X
Berit (pensioner)	X		X	
Charlotte (pensioner)		X	X	
Daniel (pensioner)		X	X	
Erik (pensioner)		X	X	
Fredrik (employed)	X		X	
Gustav (employed)	X		X	
Hilmer (employed)	X		X	
Jennifer (employed)	X			X
Lars (employed)	X			X
Nicklas (pensioner)	X			X
Per-Arne (pensioner)	X			X
Rebecca (employed)	X			X
Svea (pensioner)		X		X
Tove (pensioner)		X		X
Ulla (employed)		X		X
Vera (employed)		X		X
Zara (employed)		X		X
Åsa (employed)	X			X
Ängla (employed)	X			X

The participants with lived experiences of dementia all had a diagnosis of dementia, but during the recruitment, we did not have access to medical records to reveal the specific type of dementia. Some participants with dementia were managing at home without formal support at the time of the interviews, and others visited a day care center or used meals on wheels. An overview of the participants and their experiences of dementia are presented in Table 1.

### Ethics

Ethical approval was obtained from the Regional Ethical Review Board in Linköping (the county of Östergötland, Sweden) with the following record reference: 2017/62–31. The informed consent process has been followed to comply with ethical procedures when interviewing people living with dementia (Dewing, 2008; Hellström et al., 2007). Written and/or verbal consent was collected from all participants, and all data were kept confidential (World Medical Association, 2013).

### Data collection

Data were collected from semi-structured individual (II) or group interviews (GIs) (Polit & Beck, 2016) conducted separately by all authors. The choice of semi-structured individual and group interviews was decided in dialog with the participants and based on their wishes. The semi-structured individual and group interviews were held in participants’ homes, at their workplaces, at libraries, or at hired venues. The group interviews included two or three participants who were work colleagues or had met at a day care center. None of the participants withdrew during the interviews.

The interviews followed an interview guide with open and probing questions. Open questions were used such as “What kind of improvements in the community would you like to see that could improve the well-being and health of people living with dementia?” and probing questions (Lincoln & Guba, 1985) such as “Could you tell me more?” The semi-structured individual and group interviews lasted between 21 and 150 minutes (mean, 42 minutes) and were audio recorded and transcribed by a certified transcriptionist.

### Data analysis

The interview transcripts were analyzed according to thematic analysis (Braun & Clarke, 2006) in line with the aim of the study. An inductive and semantic approach was used to identify the themes related to the experiences of the participants. During the analysis, all transcripts had the same value and a unique voice. The data analysis was directed by six phases (Braun & Clarke, 2006). In the first phase of the analysis, the transcripts were reread numerous times and notes were taken in order to become familiar with the data. In the second phase of the analysis, the data from the transcripts were coded inductively from the bottom up to capture the experiences on a semantic level. Here, the coding was done separately by the first and last authors. In the third phase, the search for subthemes began by gathering all codes to achieve a common overview of the subthemes. In the fourth phase, the subthemes from the previous phase were revised and developed in relation to the whole dataset and the coded extracts by writing down potential subthemes on a whiteboard. An overview of the subthemes was then completed. In the fifth phase, an ongoing process involved naming the subthemes and identifying meanings that would describe the overall story of the data; the main themes were established in this phase. The subthemes and main themes were discussed by all authors to ensure the trustworthiness of the themes and attain validity (Nowell et al., 2017). In the sixth phase, the analysis was presented according to the main themes in the Findings section.

## Findings

The analysis of the interview transcripts identified four main themes: health promotion through knowledge and public awareness, health promotion through opportunities to be active, health promotion through meaningful meeting places, and health promotion through improvements in the welfare system.

### Health promotion through knowledge and public awareness

The first theme concerns the low level of knowledge and public awareness about dementia and health promotion for people with dementia in all sectors of services at individual, group, and societal levels in the municipality. The participants with lived experiences of dementia expressed that preconceived ideas about dementia in general strongly influenced them in a negative sense in that they were often ignored and not respected as active citizens with their own resources in the local community. For example, Astrid, one the participants, expressed how often people she meets talk about her dementia diagnosis and do not focus on her as a whole person:

I feel in general that people must respect us a little more, we are not stupid… people with dementia can speak up for themselves… Just treat us for the person we are, not for our condition. (Astrid, II)

These preconceived notions and attitudes about dementia as a diagnosis were also shared by other participants. To increase the knowledge about dementia, participants suggested the need for public education about dementia in the community for all ages. Tove shared her experiences as a voluntary employee, after meeting a woman with dementia living at home who had stopped going out and shopping for some days because of the lack of knowledge about dementia and after treatment from a cashier when she said that she had dementia:

I have Alzheimer’s,’ she said. Then the cashier at the shopping mall said: “Well in general all of us have that.” The treatment from the cashier made this person very sad and for days she didn’t want to go out. (Tove, GI)

Participants emphasized the importance of interpersonal relationships between people in the local community to build more knowledge and awareness about how people living with dementia want to be treated. Many of the participants expressed that ignorance in the local community would then decrease, and the community could become more inclusive and focus on a health-promoting perspective for all, not just for those living with dementia. Carers, professionals, and voluntary employees expressed that the local community underestimated the capacity of people living with dementia and did not see the effects of health promotion. They embraced the power of supporting their own resources as assets of people living with dementia rather than first seeing the diagnosis of dementia. This approach was also requested in the social and healthcare systems to include more patience and knowledge in dialog with people with dementia. Our participants discussed different strategies for how awareness of dementia could increase through exhibitions and workshops about dementia in local public spaces. Another idea that was suggested was to assign a support worker to people with dementia who they could call if they needed help or to guide them to find strategies to promote their health from the first day after being diagnosed.

### Health promotion through opportunities to be active

The second theme that many of our interviewees with different experiences of dementia discussed with us was the importance of having opportunities to be active near home as a way of improving health and well-being. Simply keeping up with the daily activities during the week, for example, by participating in a theater group or cycling to the supermarket, was expressed as strategies to take part in activities to maintain health and well-being. For instance, for Berit, one of the participants with dementia who lived in the countryside, it was the trip to gymnastics provided by the nearest municipality that kept her active and gave her opportunities to feel well:

… In the morning, the first activity is to go shopping. Later, it’s time for some gymnastics (Keep Fit) where we sit on a chair and do some exercises, and then gymnastics again in the afternoon, so Tuesday is booked. (Berit, GI)

Not only participants with dementia expressed the value of having opportunities for daily activities. Jennifer, a carer for her mother with dementia, had her own experiences to share:

… To have opportunities to be active... Physical activities are very important for those living with dementia who have the capacity to participate in these activities. (Jennifer, II)

For others, walks in the neighborhood promoted their well-being and health by being outdoors. Some of the participants with professional experiences expressed that people living with dementia at home could be granted help from home care staff to take them out for a walk, but the time set for the walks has recently been reduced through a new regulation in the community. Ulla (care staff) shared with us how much one of her patients with dementia appreciated these walks, but the reduced time was not adjusted according to his wishes:

He is almost 90 years old and has only 30 minutes for his walks, because his time for walks has been reduced from 1 hour. Even if we say ‘You know, you have only half the time now,’ he cannot walk faster. He needs his walks as he lives for them every week. (Ulla, GI)

Being able to spend time outside the home and doing daily activities when needed and at a chosen time were referred to by participants as promoting health and well-being when living with dementia. This was associated with the freedom of being independent and the sense of self-efficiency represented by walking and being active together with others as ways of getting control in everyday life with dementia.

### Health promotion through meaningful meeting places

The participants described how important social participation in public meeting places, for example, libraries or parks, was to maintain social involvement in the community for those living with dementia. The participants with lived experiences of dementia expressed how difficult it was for them to find meaningful meeting places out in the wider local community.

Rebecca, a social worker, expressed how meeting places provided by the municipality for older people in the community were closing down. This led to a negative spiral where the rights of people with dementia as citizens to participate and be part of the community were not considered. Rebecca highlighted that more efforts have to be made to break this spiral in the local community to have meaningful and inclusive meeting places in public spaces where people with dementia can meet others:

Instead of [the municipality] closing every open meeting place [in public spaces], people with dementia should be given opportunities to come out into the neighborhood and be able to use public places to meet other people. (Rebecca, II)

This development of reducing publicly funded public spaces, such as open meeting places for older people, means that people with dementia have to rely on others to help them to participate in activities. For Charlotte, most of her activities in the local community were with her partner, who arranged these events as a couple. Charlotte expressed how she loved to participate in activities along with her partner that she had never had done before, such as playing golf for first time in her life. Thanks to her partner, Charlotte was an active citizen and took part in the daily activities that were offered in the community.

Nonetheless, the participants with lived experiences of dementia shared with us that they were not invited to discussions or meetings on a local community level where could have a chance to express their feelings, for example, about shutting down the local meeting places. Astrid expressed how she missed participating in discussions with stakeholders in the community since she was diagnosed with dementia, for example, when the local meeting place that was valuable for her well-being and health was closed:

One should have the possibility to participate and discuss. I enjoy debates and everything like that. There is never something like that, and I miss it a bit, to be accepted as one of the group. (Astrid, II)

As Astrid expressed, there were few public meeting places in the local community where people with dementia could participate beyond the places provided by health and social care. People living with dementia did not have the same options to choose meaningful meeting places in public spaces as they could before the condition was diagnosed. One of the participants, Zara, suggested how new forms of places in the community could be established to encourage social connectedness:

But some form of open activity or social meeting place or something that is open to all despite diagnosis or age should be offered to the citizens in this community… (Zara, GI)

These activity areas should be new public meeting places that could be run by volunteers and not only by municipalities, where different generations could meet and where new social contacts could be formed. Beyond these public places, the participants with dementia also expressed the need for more benches in public spaces that could lead to new social contacts. Benches have an important role in encouraging people to venture out of their home and form new social engagement between citizens (Ottoni et al., 2016). Being socially active supported the health and well-being of people with dementia. Several suggestions on developing a more inclusive community were brought up, such as having access to meeting places near their own neighborhood that were open and inclusive to all. These places could increase social interactions and understanding of dementia from those with lived experiences of dementia.

### Health promotion through improvements in the welfare system

The Swedish welfare system includes many different authorities, organizations, voluntary organizations, and social care in the municipalities such as day care centers. The day care center was described as one of the places that improved their well-being (Odzakovic et al., 2018) just by being surrounded by others and to feel a sense of coherence. For Fredrik, the day care center was essential in his everyday life to break his solitude as a new resident in the community. Here, he could create new social contacts:

There are different activities, but it’s very good for those of us who live alone and don’t know anyone here (in the city) and don’t have children nearby. I enjoy being at the day care center. There are nice people here, both staff and the others. (Fredrik, II)

However, participants with different experiences of dementia expressed how day care centers and teams specializing in dementia needed to work more toward person-centered care and health-promoting improvements. They addressed that the voices and abilities of people with lived experiences of dementia have to be central and retained in the welfare system and in relation to planning for inclusive dementia-friendly communities. Some participants with different experiences of dementia talked about changes they wanted to see; for example, at day care centers, there should be more focus on the interests and strengths of people with dementia and more on health-promoting improvements. People with lived experiences of dementia expressed that they wanted to take part in the planning of, for example, daily activities at the day care centers or in dementia care practices. Per-Arne spoke about his experiences of caring for his wife with dementia when home care services appeared in their home, and the value of staff continuity: *“That is continuity as a whole. It’s important that she knows and recognizes people… Yes, it works really well.”* However, Vera, who works in home care services, expressed how the time for each home care visit had been greatly reduced, which limited their ability to work according to a health-promoting approach:

All patients have had their time reduced [by the municipality]. But it is not specified precisely for people with dementia; some patients with dementia do not even register that we have entered their home until we are gone. (Vera, GI)

These changes in dementia care practices along with underdiagnosis of dementia were discussed by many participants. Beatrice, with professional experiences of dementia, expressed the need for a health-promoting approach in the welfare system because some of her patients with dementia lived for many years in their own home without a dementia diagnosis and without any support:

It takes many years for a person to be diagnosed with dementia. Therefore, they have to be acknowledged by the health care system in some way because many patients with dementia do not need care at the pre-diagnostic stage. We need to be prepared for a more health-promoting approach because people with dementia will live for a longer period of time with dementia in our society. (Beatrice, II)

In addition to Beatrice’s experiences, carers, professionals, and voluntary employees also expressed that dementia care practices were working on one aspect of the problem and did not see the whole person in context from a health-promoting perspective. The underlying problem according to the participants working in dementia care practices was that the welfare system was not prepared or did not have the resources to help people living with dementia at home. Broader cooperation between the authorities, stakeholders, and people with dementia with a focus on the needs and wishes of the person with lived experiences of dementia was requested in order to develop an inclusive community.

## Discussion

Health-promoting strategies have been shown to be sought after by people with lived, professional, and personal experiences of dementia, striving to live well after getting a dementia diagnosis. To self-regulate and to have the possibility to be outdoors and to socialize with others in meeting places in public spaces are essential in everyday life for people living with dementia. Nevertheless, knowledge and public awareness of dementia must be increased in the community at individual, group, and societal levels in order to make dementia a public health issue. We found that the need for dementia literacy was great and removing the stigmatization of dementia as a hidden condition has to be a goal for interventions in society.

Swaffer (2014) argued that there is a need worldwide to address dementia as a global public health issue and that has to start with social action in local communities where people with dementia are engaged; in that way, the stigma and social isolation can be reduced. The development of dementia-friendly communities has thus far been outlined in a paternalistic way, with people other than those with dementia themselves involved as experts. As Donnelly et al. (2018) discussed, family carers were often listened to first when making decisions about the care of people with dementia. Unfortunately, the voices and initiatives of the people living with dementia themselves are often not heard when developing dementia-friendly communities (Milne, 2010; Swaffer, 2014). We have involved people with different experiences of dementia as our experts to inform us how they want an inclusive dementia-friendly community to be. Our participants expressed how citizens in the municipality and neighbors had no knowledge or awareness about how everyday life could be when living with dementia. If we want to develop dementia-friendly communities that are inclusive, then people with dementia have to be included in this process (Herbert & Scales, 2019; Heward et al., 2017). They have to be included as active agents of their lives, just like anyone else in a community (Nedlund & Bartlett, 2017). Therefore, a more focused citizenship agenda can improve the lives and treatment of those living with dementia within the community and dementia care practice (Bartlett & O´Connor, 2007).

Our findings show that knowledge and awareness about dementia are essential, both within the municipality and in society as a whole. Dementia as a condition is often seen as a universal disease and something that the participants we spoke to had to face within their everyday life. The participants with dementia expressed how they had to find their own strategies to maintain their health and well-being or rely on others to do the things that were important to them, which goes beyond the biomedical approach. Still, the biomedical approach to dementia exists, and we spoke to people who had different experiences of dementia. They expressed that attitudes to dementia have to change, and public education at all levels in society is necessary. It was requested that knowledge about dementia should be deepened and spread more widely within society and by those living with dementia. The importance of public awareness about dementia when achieving dementia-friendly communities (Alzheimer’s Disease International, 2016; Cahill, 2015; Vernooij-Dassen et al., 2005) was in line with our findings; our participants requested a need for public education for all citizens if the municipality wanted to develop a dementia-friendly community. Several models around the world have been devised to support the establishment of dementia-friendly communities aimed at public awareness campaigns about dementia, for example, in the United Kingdom (Crampton & Eley, 2013) and Australia (Phillipson et al., 2018; Courtney-Pratt et al., 2018). Education about dementia for children of all ages (Baker et al., 2018) and undergraduate healthcare professionals (Cashin et al., 2018) is one of the first steps needed to reduce the stigma related to dementia. Using online newspapers to inform citizens about dementia (Werner et al., 2017) could also spread knowledge and awareness of dementia. In line with this, there is a need for a national campaign in Sweden (Ministry of Health and Social Affairs, 2018) or a public exhibition to raise awareness about dementia, and this should be included in the framework of dementia-friendly communities, not only from a Swedish perspective but also worldwide. Hence, this study contributes with knowledge about how to develop dementia-friendly communities with a health-promoting perspective that could enrich the national strategy for dementia in Sweden (Ministry of Health and Social Affairs, 2018).

Looking beyond questions of knowledge and awareness of dementia, this study underlines the importance of health promotion through opportunities to be active, together with others, strengthen well-being, and experience health among people living with dementia. There is often a tendency to highlight deficiencies rather than the strengths of people with dementia (Bartlett, 2016; Henwood & Downs, 2014; Phillpson et al., 2018; Rodgers, 2018). This phenomenon of discussing or defining people based only on limitations seems to be more common when it comes to people with dementia compared with other conditions. Swaffer (2015) identified how dementia was the only disease or condition where both those living with the condition and their families were told to go home and give up their lives instead of fighting for their future well-being. When people with dementia are given a voice and the same right to be visible in the community as other people living with long-term conditions, this will be the first step toward raising public awareness of dementia as a public health issue.

Wiersma and Denton (2016) and Clark et al. (2020) have also highlighted the importance of having social support, where people care for and look after each other, as a key attribute of creating social networks in the community. This will become a priority as people age at home as long as they can (Rowles, 1981) and remain connected to their communities. Hence, the importance of health inequalities could then be decreased by establishing “healthy and sustainable places and communities” (Marmot, 2015) for every citizen, whatever their condition. Therefore, our participants expressed the value of having opportunities to promote their health through meaningful meeting places where they could meet people and participate in the community. The relationship between people and their communities creates social networks and a sense of belonging that enhances citizenship (Nedlund et al., 2019; Rahman & Swaffer, 2018). Our findings have shown that there were few opportunities for social inclusion in the community because the meeting places arranged by the welfare system had been closed for financial reasons. This insight has to be acknowledged by the national strategy plan for dementia and policymakers. Meeting places in public spaces are needed for people with dementia, as well as open activities, where all inhabitants can meet and share their understanding in order to reduce the stigma and fear about dementia. Being in a social context and living an active life have been found to be associated with social health (Cashin et al., 2018; Vernooij-Dassen & Jeon, 2016; Ward et al., 2018) and well-being for people with dementia (Odzakovic et al., 2018). This is in line with earlier results from Rahman and Swaffer (2018) showing that health and living well with dementia were related to each another in more than one way. This approach has to be incorporated more into the key principles of dementia-friendly communities by policymakers and professionals in dementia care practice. Policymakers have to involve people with dementia as educators when developing standards for planning home care services and social activities in communities. A support worker could be assigned directly when a person gets a dementia diagnosis, as is the case in Scotland (The Scottish Government, 2017), to support them throughout the fragmented welfare system and to improve their well-being during the post-diagnosis stage. It is suggested that the professionals working in municipality home care services could take on the role of providing support in Sweden, if they receive resources. Also, the role of professionals in care practice has to be acknowledged when building up dementia-friendly communities (Rahman & Swaffer, 2018; Shannon et al., 2018).

### Limitations and strengths

The setting for this study was drawn from a Swedish context where there may be cultural nuances and norms that could differ from other countries and could limit the transferability of our data to other contexts. Despite this limitation, it has been shown that initiatives to establish dementia-friendly communities built on a health promotion approach have to start at a local level in a community and then hopefully aspire to a national level (Alzheimer’s Disease International, 2016). An additional limitation is that during the recruitment of participants, we became aware that some gatekeepers hesitated to ask people with dementia if they wanted to participate in our study. These situations could explain why there were some difficulties in recruiting people with dementia from the municipality to participate in this study. This explains our small number of people living with dementia, which reduces the credibility and transferability of the findings. Finally, the use of both individual and group interviews could be a limitation in relation to participants’ responses. However, the choice of participating in a group interview was based on the wishes of the participants because this type of interview situation was most comfortable for them (Phillipson & Hammond, 2018).

Beyond these limitations, the strengths of this study are that all authors were involved in creating an interview guide and conducting the interviews. All voices and experiences of dementia were treated as equal during the analysis, and this was another strength of the study. This study is unique; there are few earlier studies addressing strategies for framing dementia-friendly communities (Shannon et al., 2018) from a health-promoting perspective and including both people with lived, personal, and professional experiences of dementia, especially in Sweden. It was not until 2018 that the first national Swedish strategy for caring for people with dementia in which dementia-friendly communities are mentioned was adopted by the government (Ministry of Health and Social Affairs, 2018), but the perspectives of people with dementia or others with experiences of dementia are missing. This study has explored different types of experiences related to dementia that could provide knowledge on structuring dementia-friendly communities internationally in a health-promoting manner where the experiences and strengths of dementia are central.

## Conclusions

Knowledge and awareness about dementia are needed from a health-promoting perspective in order to increase the prominence of dementia as a public health issue. The existing literature documents that involvement of people living with dementia (Herbert & Scales, 2019; Heward et al., 2017; Swaffer, 2014) and public education on dementia for all ages are necessary when developing dementia-friendly communities (Baker et al., 2018; Cashin et al., 2018; Shannon et al., 2018). This study contributes to the existing literature by showing the need for health-promoting actions at individual, group, and community levels. People with dementia have to be seen as active agents of their own life. Society has to arrange supportive environments where people with dementia can be included and to support people to maintain their well-being and health. It is time to see beyond the condition, as for other long-term conditions, and to work toward inclusive communities where health and well-being are considered for all equally. In this study, we have identified how different strategies could embrace awareness about dementia in public outdoor spaces. Despite national strategies for dementia worldwide, little attention has been paid to how people with dementia can live well with dementia and what strengths they have to embrace their health. However, much research on other chronic conditions has focused on a health-promoting perspective but not when it comes to dementia. Hence, this study can contribute to inform the emergence of dementia-friendly communities (Alzheimer’s Disease International, 2016; Ministry of Health and Social Affairs, 2018) worldwide. Finally, further research should focus more on the strengths of people with dementia and how professionals in dementia care practice could be part of promoting health through a salutogenic approach.
